# Boron Nitride/Carbon Fiber High-Oriented Thermal Conductivity Material with Leaves–Branches Structure

**DOI:** 10.3390/ma17102183

**Published:** 2024-05-07

**Authors:** Dengfeng Shu, Jiachen Sun, Fei Huang, Wenbo Qin, Chengbiao Wang, Wen Yue

**Affiliations:** 1 HYMN Advance Materials Technology (Shenzhen), Shenzhen 518000, China; shu_scaler@163.com (D.S.); 15262596669@163.com (J.S.); huangfei66@outlook.com (F.H.); cbwang@cugb.edu.cn (C.W.); 2 School of Engineering and Technology, China University of Geosciences (Beijing), Beijing 100083, China; cugbyw@163.com; 3 Zhengzhou Institute, China University of Geosciences (Beijing), Zhengzhou 451283, China

**Keywords:** structural, interfaces, polymers, thermal properties, carbon fibers, boron nitride

## Abstract

In the realm of thermal interface materials (TIMs), high thermal conductivity and low density are key for effective thermal management and are particularly vital due to the growing compactness and lightweight nature of electronic devices. Efficient directional arrangement is a key control strategy to significantly improve thermal conductivity and comprehensive properties of thermal interface materials. In the present work, drawing inspiration from natural leaf and branch structures, a simple-to-implement approach for fabricating oriented thermal conductivity composites is introduced. Utilizing carbon fibers (CFs), known for their ultra-high thermal conductivity, as branches, this design ensures robust thermal conduction channels. Concurrently, boron nitride (BN) platelets, characterized by their substantial in-plane thermal conductivity, act as leaves. These components not only support the branches but also serve as junctions in the thermal conduction network. Remarkably, the composite achieves a thermal conductivity of 11.08 W/(m·K) with just an 11.1 wt% CF content and a 1.86 g/cm^3^ density. This study expands the methodologies for achieving highly oriented configurations of fibrous and flake materials, which provides a new design idea for preparing high-thermal conductivity and low-density thermal interface materials.

## 1. Introduction

The evolution of electronic devices, including Insulated Gate Bipolar Transistors (IGBTs), Optical Modules, 5G Base Stations, and Vehicle Controllers, has steered electronic components towards more compact sizes, higher integration, and increased power. While electronic equipment has improved in performance, there has been a significant increase in heat density. If this heat is not properly dissipated, it can have a negative impact on a device’s performance, stability, and overall service life. TIMs serve as a crucial link between the heat source and the heat sink, facilitating efficient heat transfer. The thermal conductivity and interfacial thermal resistance of TIMs are the key indicators of their performance. Some previous studies reported that interfacial thermal resistance is controlled by the overlapping of phonon density-of-states and group velocity similarity [1,2]. Through theoretical calculations and experiments, interface thermal resistance has been effectively reduced. Therefore, this shift imposes greater demands on the thermal conductivity capabilities of thermal interface materials (TIMs) [3,4,5,6]. Silicone rubber (SIR) is widely selected as the matrix material for TIMs due to its wide viscosity range, adjustable hardness, high insulation, good filler compatibility, and excellent reliability [7,8,9]. Compared with polyurethane, acrylic acid, and epoxy resin, SIR has better filling ability and reliability with respect to thermally conductive particles. These advantages make SIR the first choice for the preparation of high-thermal conductivity TIM materials [10,11]. Traditional TIMs have attempted to enhance thermal conductivity by incorporating isotropic ceramic particles (such as alumina and silicon carbide), a method with limited effectiveness as per thermal percolation theory [12,13,14]. High-thermal conductivity materials like CF and carbon nanotubes (one-dimensional (1D) materials), along with BN and graphene (two-dimensional (2D) materials), exhibit anisotropic properties [15,16]. CF is widely used in the design of orientation structures because of its excellent thermal conductivity, electrical, and magnetic properties. BN is known as white graphite because of its excellent properties and has a thermal conductivity of 200–600 W/(m·K). The unique insulating sheet structure makes it widely selected in thermal interface materials with directional structure.

To exploit these properties, researchers have manipulated material orientation in TIMs using mechanical [17,18], electrical [19,20], and magnetic methods [21,22], aiming to boost thermal conductivity. Huang et al. [17] used a syringe to orient a CF/alumina composite material, forcing the CF to form a regular orientation arrangement inside the material by limiting the flow of the material in a slender tube. The influence of different contents of CF on the thermal conductivity was explored. By analyzing the internal microstructure of the material, it was concluded that the thermal conductivity increases first and then decreases with the increase in CF content. The highest thermal conductivity reached 21.29 W/(m·K) when the CF content was 12.1 wt%. Alumina provided support and shear force for CF and provided a speed difference for CF in the process of orientation. The deflection of CF in the flow process was suppressed. This method is convenient and easy to implement, but it is difficult to prepare material in batches. Niu et al. [18] constructed a vertical arrangement structure of anisotropic fillers in composite materials with the help of expansion flow. Using the shear motion of the composite material in the narrow channel into the wide channel, the orientation of BN was realized by adjusting the size of the channel and controlling the shear rate and expansion rate. The BN sheet in the silicone gel strip was oriented into a curved shape, which included vertical alignment in the central region and horizontal alignment near the strip surface. Due to the vertical orientation of BN in the central region of the strip, a planar thermal conductivity of up to 5.65 W/m·K was obtained. Further, CF was used to enhance the thermal conductivity. Combining BN with pitch-based carbon fiber, it could be further improved to reach 6.54 W/m·K. However, the center and edge orientation effects of the method are inconsistent, which means that there are obstacles to its practical application. By anchoring a special structure on the surface of the filler, cho et al. used an electric field to orient the anisotropic material [19]. By changing the electric field conditions and BN content, BN was successfully aligned. The effect of the aspect ratio of BN and the increase in the load level on the thermal conductivity was investigated. The thermal conductivity reached 1.56 W/m·K when the BN composite reached 15 vol%. In the case of maintaining high thermal conductivity, the material itself remains insulating, which promotes practical applications. However, the preparation steps for directional materials by electric fields are complicated, such that they are not conducive to further application. Li et al. [20] used the electrostatic flocking assembly strategy to construct a neatly arranged BN structure network in several continuous layers, thereby improving the thermal conductivity of composites. The effect of different amounts of BN on the thermal conductivity of BN/epoxy resin composites was investigated. Under the highest BN loading of 17.6 wt%, the thermal conductivity of BN/epoxy film composites reached 0.65 W/m·K, which was 18.6% and 204% higher than that of random BN/polymer (0.549 W/m·K) and pure epoxy resin (0.214 W/m·K), respectively. In addition, the BN/epoxy resin film exhibited good elastic properties and tensile strength. The researchers studied the thermal conductivity enhancement of carbon-based composite TIMs by using a 10 T superconducting magnet without low temperature to arrange the graphite fillers [21]. The graphite suspended in the polymer solution was perpendicular to the magnetic field orientation because the graphite had a great anisotropy in magnetic susceptibility. At 80 wt% filler loading, it showed a 330% increase in thermal conductivity. The results showed that the increase in thermal conductivity anisotropy was related to the better packing arrangement at a high filler content. Yuan et al. [22] used FeCo nanocubes as an auxiliary to help BN nanosheets arrange vertically to form a good heat conduction channel. The orientation of the composites in PDMS was controlled by an external magnetic field. A structure with a large size range, high density, and high vertical orientation was successfully obtained. The maximum thermal conductivity of the composite film was 2.25 W/m·K, which was about seven times that of the composite film containing BN. However, these techniques often involve single-type fillers, complex processes, and high energy consumption. Inspired by leaf and branch structures, this article integrates 1D and 2D materials, effectively harnessing the longitudinal thermal conductivity of fiber materials and the in-plane thermal conductivity of sheet materials.

In this paper, inspired by the structure of leaves and branches, a structure for a directional high-thermal conductivity composite material which can be prepared by simple extrusion molding is proposed. The study focuses on orienting BN and CF into a highly aligned structure in the XY orientation within a silicone rubber matrix by minimizing gaps during the extrusion process. Through small-size extrusion molding, CF and BN become highly oriented structures in the silicone rubber matrix. CF with ultra-high thermal conductivity provides excellent thermal conduction paths as branches, and flake BN with significant in-plane thermal conductivity provides sufficient support for branches as leaves and serves as a series connection of thermal conduction paths. It not only reduces the filling amount of CF and reduces the cost, but also achieves large-area thermal conductivity through low-density BN. Further, the addition of low-density CF and BN reduces the density of TIMs with the aim of achieving high thermal conductivity. To achieve excellent directional effect, the gap during the extrusion process was set to be close to the length of the CF. To validate the experimental findings, BN/CF composites with both random and oriented distributions were compared, highlighting the benefits of the BN/CF combination over CF alone.

## 2. Materials and Methods

### 2.1. Materials

Commercially available CF was purchased from Denka Co., Ltd. (Tokyo, Japan), characterized by dimensions of 6–8 μm in diameter and 250–300 μm in length and a high thermal conductivity of 1500 W/(m⋅K). BN platelets, with a diameter of 30 μm, a thickness ranging between 1 and 3 μm, and an in-plane thermal conductivity of 300 W/(m·K), were obtained from Bestry Performance Materials Co., Ltd. (Shanghai, China). Spherical alumina with a median particle size of approximately 30 μm and a thermal conductivity of 30 W/(m·K) was also obtained from the same supplier. The liquid SIR matrix (Additive Silicone Rubber Model CX3151) was acquired from Guangdong Trancy New Materials Technology Co., Ltd. (Foshan, China). Component A included vinyl silicone oil and Karstedt catalyst. Component B included vinyl silicone oil, hydrogen silicone oil, and ethynyl cyclohexanol inhibitor. The two components of the rubber were mixed in a 1:1 ratio immediately prior to use, ensuring a homogeneous blend for the composite material preparation.

### 2.2. Preparation of Silicone Rubber Matrix and Random Composite Silicone Rubber

All materials were weighed and added to the mixing tank according to the proportions set for each component in the matrix and random group in Table 1. To ensure the accuracy of weighing, an electronic balance with an accuracy of 0.001 g was used. The mixing tank was placed in a vacuum mixer and run for 10 min. The stirring speed was set to 500 rpm. During the stirring process, the vacuum pump was turned on to remove air bubbles in the slurry. A release film was placed in the mold to form a flat surface. The prepared slurry was transferred to the mold and heated. The heating temperature was set to 120 °C, and the heating time was 30 min. After heating, the slurry was placed in a drying oven until room temperature and taken out. The release film was peeled off to obtain the Random Composite Silicone Rubber. The experimental groups were as follows: Matrix, R-BN, R-BN/CF-1, R-BN/CF-2, and R-BN/CF-3.

### 2.3. Preparation of Oriented Composite Silicone Rubber

**Figure 1:**
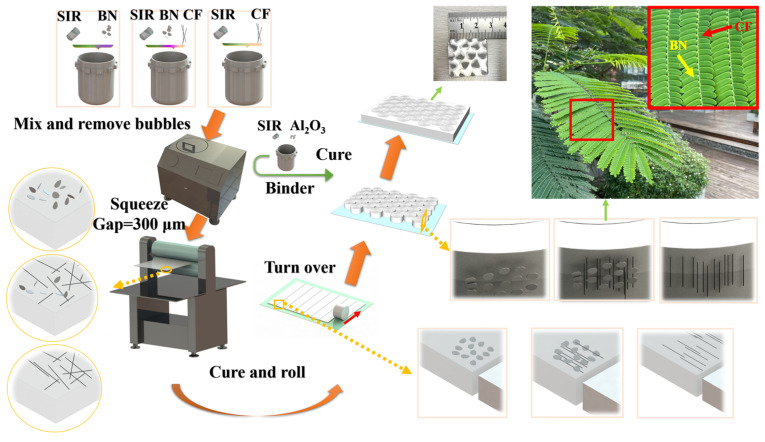
Preparation process diagram of oriented composites. The internal schematics of different preparation stages are displayed. The final internal structure is similar to the natural leaves–branches structure.

All materials were accurately measured according to the predetermined ratios for each component in the oriented group, as outlined in Table 1 and Figure 1. To ensure the accuracy of weighing, an electronic balance with an accuracy of 0.001 g was used. These materials were then transferred to a mixing tank for thorough dispersion and bubble elimination. The stirring time was set to 10 min, and the stirring speed was set to 500 rpm. Following dispersion, the slurry was placed on the release film, a vacuum pump was used to remove air bubbles generated during the placement of the slurry, and a layer of release film was covered on the slurry. The combined materials underwent an extrusion process using a double roll machine set at a 300 μm gap and an extrusion rate of 1 mm/s. The disordered BN and CF in the slurry experienced a sudden decrease in Z-direction size, resulting in a large shear effect. In the process of passing through the gap, BN increased the shear rate, forcing CF to move along the axial direction. At the same time, the lamellar BN also formed a directional arrangement because the movement in the Z direction was limited. After rolling, the interior of the composite material was arranged in a regular arrangement of BN and CF. After extrusion, the materials were subjected to a heating and curing process. The heating temperature was set to 120 °C, and the heating time was 30 min. After the heating was completed, the combined materials were placed in a drying box and cooled to room temperature. The release film was removed to obtain the cured silicone rubber. The BN/CF oriented structure was fixed inside the composite material, and the orientation direction was horizontal. The resultant cured silicone rubber was then sliced perpendicularly to the extrusion orientation. These slices were subsequently rolled into cylinders aligned with the cutting orientation. The formed small units were carefully flipped and arranged with their cross-sections facing upwards. Due to the reversal of the direction, the arrangement direction of the BN/CF changed to the vertical direction. The gaps in these assemblies were filled with a binder, followed by a secondary phase of heating and curing. The heating temperature was set to 120 °C, and the heating time was 30 min. During the curing process, a release film was used to achieve the purpose of forming a flat surface. The use of the binder made the directional unit fixed and improved the flexibility of the composite material. This process yielded oriented silicone rubber materials, specifically O-BN, O-BN/CF-1, O-BN/CF-2, O-BN/CF-3, O-CF-1, O-CF-2, and O-CF-3.

### 2.4. Preparation of Binder

The silicone rubber matrix was considered a suitable binder for fixing and combining directional units. By adding spherical alumina to molded silicone rubber, the thermal conductivity of silicone rubber can be improved, which can prevent the influence of a small amount of curing agent covering the vertical plane [17]. Each material was precisely weighed and placed into a mixing tank in accordance with the specific ratios designated for each component in Table 1. The mixture underwent thorough dispersion and bubble removal to create the binder. It is worth noting that the binder needs to be at room temperature or below room temperature during the preparation and preservation process to avoid premature curing and failure of the binder due to excessive temperature.

### 2.5. Test and Characterization

Thermal conductivity measurements were conducted using a TIM thermal resistance and conductivity tester (Longwin 9389, Taoyuan, China). The hot end temperature of the tester was set to 80 °C, and the cold end was set to 30 °C. To simulate the case of TIMs, test criteria with loads were used (viscoelastic solids for which stresses of deformation were ultimately balanced by internal material stresses, thus limiting further deformation). The test pressure was set to 20 Psi. All samples were prepared in three thicknesses of 1 mm, 2 mm, and 3 mm. Scanning Electron Microscopy (SEM, ZEISS Gemini 300, Jena, Germany) was employed to examine both the initial morphology of the materials and the cross-sections of the composites. The arrangement of BN and CF in the interior was displayed by SEM, and the synergistic effect between the fillers and the effect of different filler contents on the orientation degree were discussed. An infrared detector (Dianytech CA-10, Dongguan, China) was utilized to assess the temperature variations in ceramic heating plates, each with a rated power of 20 W, when applied to different composites. In order to eliminate the differences in contact thermal resistance between different types of composite materials, thermal silicone grease (TSG-400) was used in the gap between composite materials, radiators, and ceramic sheets. The TSG-400 was purchased from HYMN Advance Material Technology Co., Ltd. (Shenzhen, China). Infrared thermal imaging can intuitively reflect the ability of TIMs to reduce chip temperature in practical applications. The Density Tester (Matsuhaku DX300, Taichung, China) was used to test the density of the composites.

## 3. Results and Discussion

### 3.1. Thermal Conductivity of Composites

**Figure 2:**
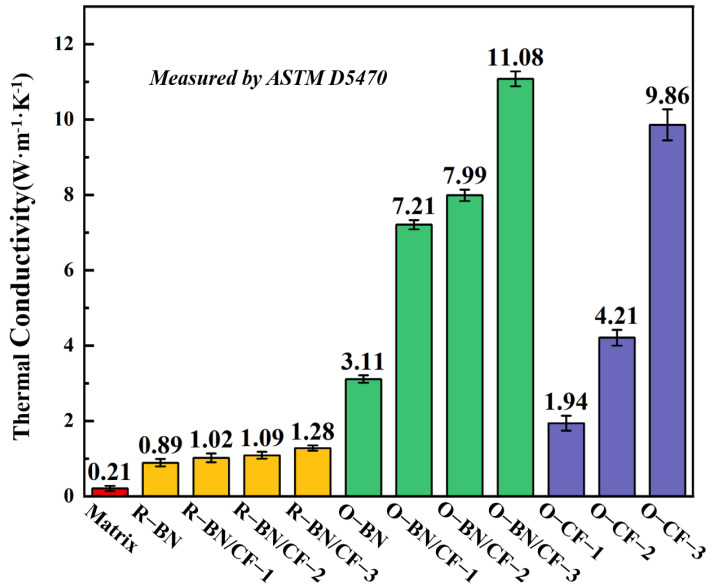
Thermal conductivity of different composites. The red represents the SIR matrix, the yellow represents the random composite material, the green represents the oriented composite material with BN components, and the blue represents the oriented composite material without BN components.

Figure 2 illustrates the thermal conductivities of various composites. As shown in the figure, the thermal conductivity of the silicone rubber matrix group is 0.21 W/(m·K), which is at a low level. The thermal conductivity increases significantly when there is randomly distributed BN in the silicone rubber. Composites with random distribution show only a slight increase in thermal conductivity upon CF addition, peaking at 1.28 W/(m·K). This indicates that merely increasing CF filler, despite its high intrinsic thermal conductivity, is insufficient for significant improvement. Compared with randomly arranged BN, the orientation of BN leads to a 349% increase in thermal conductivity, The regular arrangement of BN improves the thermal conductivity, allowing BN to further enhance the thermal conductivity of the composite material while providing support. For composites incorporating BN/CF, thermal conductivities escalate by 707%, 733%, and 866% for fill ratios of 4 wt.%, 7.7 wt.%, and 11.1 wt.%, respectively, achieving a maximum of 11.08 W/(m·K). To explore the advantages of BN, BN was removed from the components and the thermal conductivity of the composite material was characterized. The content of CF increased sequentially to 7.7 wt%, 14.3 wt%, and 20 wt%. On the contrary, the thermal conductivity decreased to 26.9%, 52.7%, and 89.0% of the original level. When BN platelets were added to the aligned CF composites, the content of carbon fibers decreased and the thermal conductivity increased. This not only improved the thermal conductivity, but also reduced the amount of CF. This suggests that BN platelets, as leaves, effectively support CF, enabling higher thermal conductivity with minimal oriented filler [18]. In addition, BN can also reduce the amount of CF per unit volume, thereby optimizing the cost of high-thermal conductivity composites, which promotes the practical application of composites.

### 3.2. Structural Characterization of Composites

**Figure 3:**
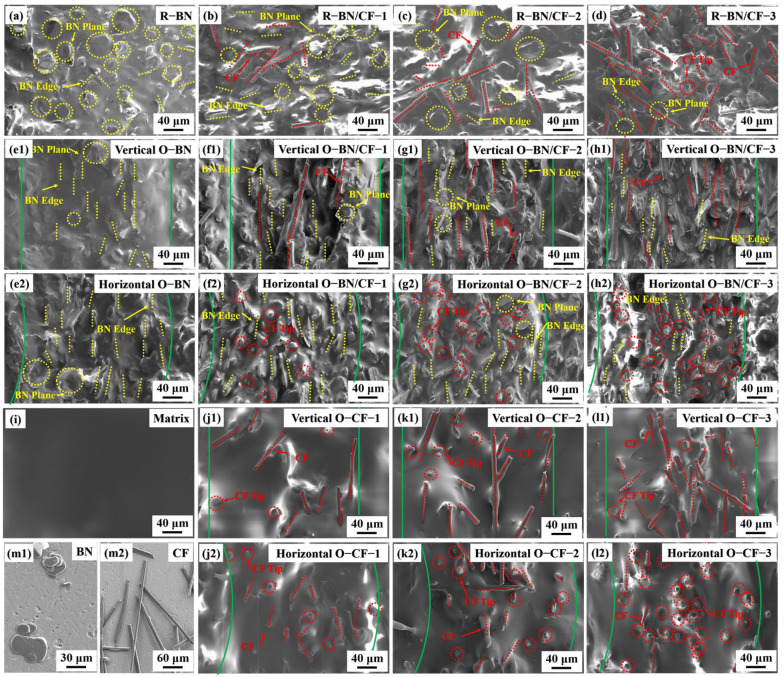
(**a**–**m2**) SEM images of longitudinal sections and cross-sections of different composite materials and SEM images of original materials. The yellow dotted lines are the BN edges, and the yellow dotted circles are the BN planes. The red dotted lines are the CF axes. The red dotted circles are the tips of CFs. The green solid lines are the boundaries of the orientation units.

To further explore the difference between the internal microstructures of the oriented composites and the random composites, the composites were cut along the horizontal and vertical directions, and the microstructures were characterized by SEM. Figure 3a shows the disordered arrangement in the cross-section of the traditional cured BN silicone rubber. The yellow dotted circles represent the state of the BN plane, and the yellow dotted straight lines represent the edge of the BN. The BN shows sheet overlap, and some BN edges are exposed, showing an irregular arrangement. Figure 3b–d show that when CF is added to the composite material, although the local thermal conduction path increases, the irregular arrangement of BN and CF leads to the local thermal conduction paths not being effectively linked together, and the limitation of thermal conductivity improvement is reflected [23]. The red dotted lines represent the CF in the axial direction, and the red dotted circles represent the tips of the CFs. Conversely, Figure 3(e1) shows mostly vertical edge orientation in oriented BN, with rare complete BN presence, indicating BN’s directionality in silicone rubber. The green solid lines in the diagram represent the boundaries of the oriented small unit. Figure 3(e2) shows the same microstructure in the horizontal section, and the edge direction is almost parallel to the vertical direction, which also verifies that BN forms a single orientation arrangement along the vertical plane inside the composites. At the same time, the directional arrangement of BN proves the effectiveness of the preparation method. CF with high thermal conductivity was added to the oriented BN composites, and the microstructure of the vertical plane is shown in Figure 3(f1,g1,h1). CF almost shows a consistent vertical distribution, and the direction is consistent with the edge of BN. With the increase in CF content, the vertical CF thermal conduction path increases, which verifies that the thermal conductivity increases with the increase in CF content. The thermal conductivity of O-BN/CF-3 was significantly improved compared to the first two groups because the content of carbon fiber reached the threshold according to the percolation threshold theory. In the vertical section, the tip of the CF was not found; on the contrary, as shown in Figure 3(f2,g2,h2), CF exists almost entirely in the horizontal section in the form of tip exposure. This indicates that CF forms a good directional arrangement inside the composite material, forming an anisotropic thermal conductive material with a high thermal conductivity path in the vertical direction. This explains the result that the thermal conductivity reached 11.08 W/(m·K) when the CF content was 11.1 wt%. Further exploration showed that the removal of BN has a great influence on the orientation arrangement of CF. Figure 3(j1,k1,l1) show that when the CF content is low, the support of BN is lost, resulting in a low degree of CF orientation and separation from each other, and the thermal conduction path is difficult to establish. When the CF content increases, the degree of orientation increases, CF produces partial contact, and the thermal conductivity is improved. This result is consistent with the law shown in Figure 3(j2,k2,l2), and the number of exposed CF tips in the horizontal plane gradually increases. This shows that when the CF content increases, a composite material with a certain orientation arrangement is formed by relying on the interaction of CF itself. With the enhancement of the directional effect and the increase in the thermal conduction path, the thermal conductivity is obviously improved. However, even when the content of CF reaches 0.2wt%, the orientation effect is still weaker than that of the BN/CF group. This leads to a lower thermal conductivity for O-CF-3 than for O-BN/CF-3, although the former has a higher CF content [17]. In particular, the group without any material addition is shown in Figure 3i, which means that there is no material inside the silicone rubber to help establish the thermal conductivity path. Figure 3(m1,m2) display the morphologies of the BN and CF used; BN exhibits a sheet structure, and the morphology of CF is fibrous.

### 3.3. Infrared Characterization of Composites

**Figure 4:**
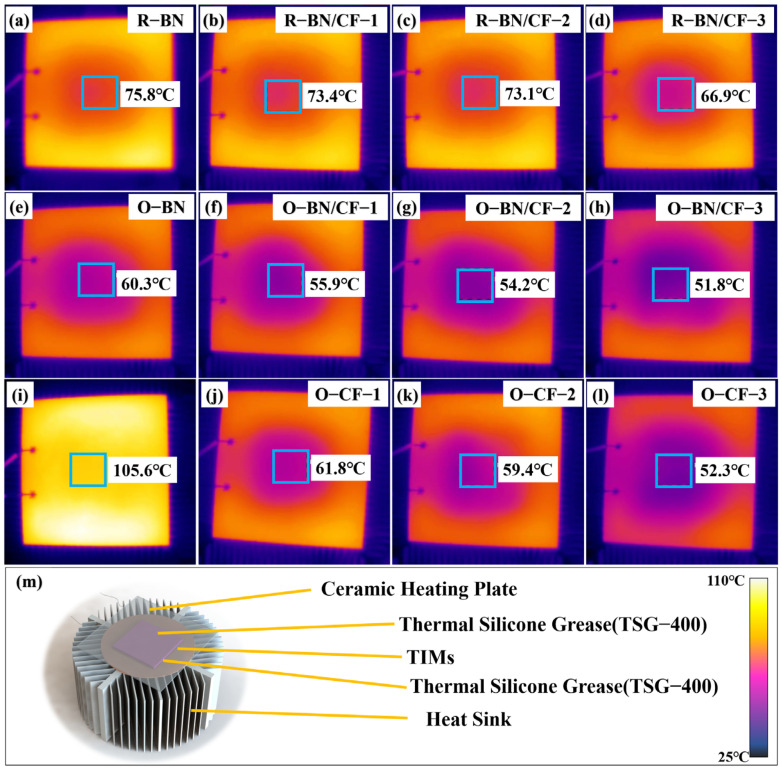
(**a**–**m**) Infrared images and experimental structural diagrams of different composites at 2 min. The temperature value is the average temperature in the box (1 cm × 1 cm).

As shown in Figure 4m, practical applications were explored by placing different composite silicone rubbers between ceramic heating plates and heat sinks. The input voltage of the ceramic heating plates was set to 20 volts, and the current was set to 1 ampere. Figure 4 shows the infrared images of the ceramic heating plate after heating for 2 min, and the final temperature of the central area is displayed in the images. To obtain more accurate test results, the temperature was set as the average temperature in the central area of the 1 cm × 1 cm plane, which avoided the problem of local temperature unevenness. TSG-400 was used to eliminate the effect of thermal contact resistance. The order of the final temperature was opposite to the order of the thermal conductivity. The larger the thermal conductivity, the smaller the final temperature, which conforms to the definition of thermal conductivity. The silicone rubber matrix group showed the highest temperature (105.6 °C). The center temperature of the random unit was at a high level, which corresponds to the low thermal conductivity, and the center temperature range of the group after the internal directional arrangement was reduced to between 51.8 °C and 61.8 °C, which shows that the directional effect on the center temperature was obvious. Among them, O-BN/CF-3 decreased to the lowest temperature of 51.8 °C. This was 15.1 °C lower than the R-BN/CF-3 group, which could significantly improve the service life of a chip. According to previous reports, for every 10 °C reduction in the temperature of a chip, the failure rate can be reduced by 50% [24].

**Figure 5:**
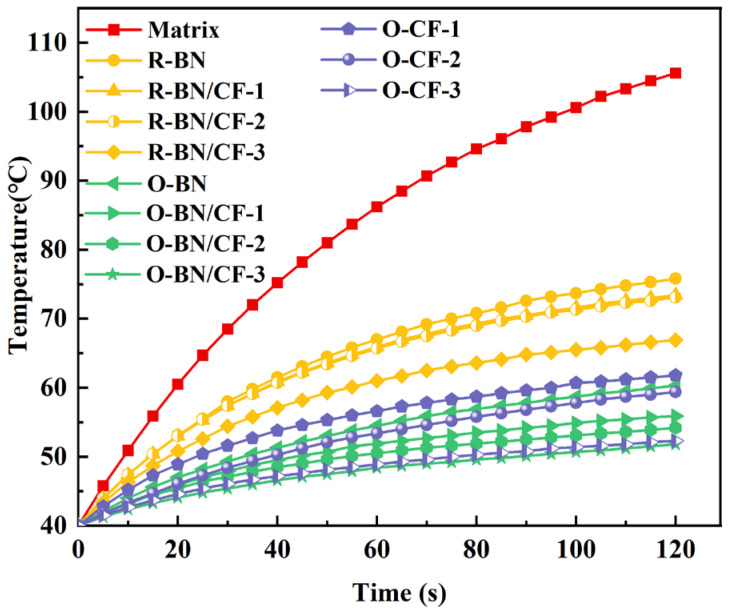
Center temperature changes of ceramic heating plates under different composites. The start temperature is 40 °C, and the time is 120 s.

Figure 5 shows the temperature rise in the ceramic heating plate’s center area over 2 min from 40 °C, confirming the positive correlation between thermal conductivity and cooling ability. The slower the rate at which the center temperature of the ceramic heating plate rises when the thermal conductivity of the TIM increases, the lower the final stable temperature, which is conducive to the stable operation of the chip.

### 3.4. Density Characterization of Composites

**Figure 6:**
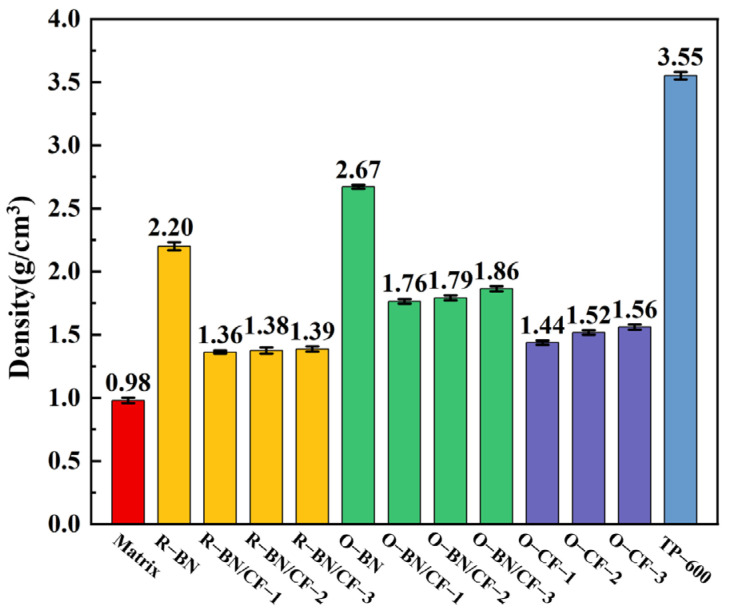
The densities of different composites. The density comparison includes the different composite materials and a commercial thermal pad.

To explore the advantages of the composites in practical applications, the density of the different composite materials was tested, and commercial 6W composite materials were selected for comparison. TP-600 was purchased from HYMN Advance Material Technology Co., Ltd., with a thermal conductivity of 6 W/(m·K). Figure 6 shows that when there is no addition inside the silicone rubber, the density reaches the lowest value, and the density of the oriented composite is higher than that of the random composites. This is because the directional composite material has a binder, and the density of the alumina particles in the binder is at a high level, resulting in a small increase in the density of the composite material. Compared with the group with BN added, the density of the BN/CF group is significantly reduced, because the density of the CF is less than that of the BN, and the use of CF could reduce the density of the composite. Furthermore, the density of O-BN/CF-3 with the highest CF content is 1.86 g/cm^3^, while the density of the control group reaches 3.55 g/cm^3^. The density of the O-BN/CF with high thermal conductivity is about half the density of the control group, which indicates that the binder will increase the density to a lesser extent, but the small amount of use has little effect on the density of the composite. The characteristics of low density can further promote the application of high-thermal conductivity composite materials in practical scenarios. From Table 1 and Figure 6, it can be seen that the density of the oriented CF group without BN is 1.56 g/cm^3^ and that the content of CF is 20 wt%. When BN is added to the oriented CF group, the density is 1.86 g/cm^3^ and the CF content is 11.1 wt%. The density increased by about 19.2%, but the CF content decreased by about 44.5%. Through calculation, it was found that when BN was added to the CF group, the weight of CF per unit volume was reduced by about 33.8%; CF being expensive, the reduction in CF content can save costs. TIMs are usually used in space-fixed environments, which means that, compared with O-CF-3, the O-BN/CF-3 group not only has a higher thermal conductivity but also a lower cost.

**Figure 7:**
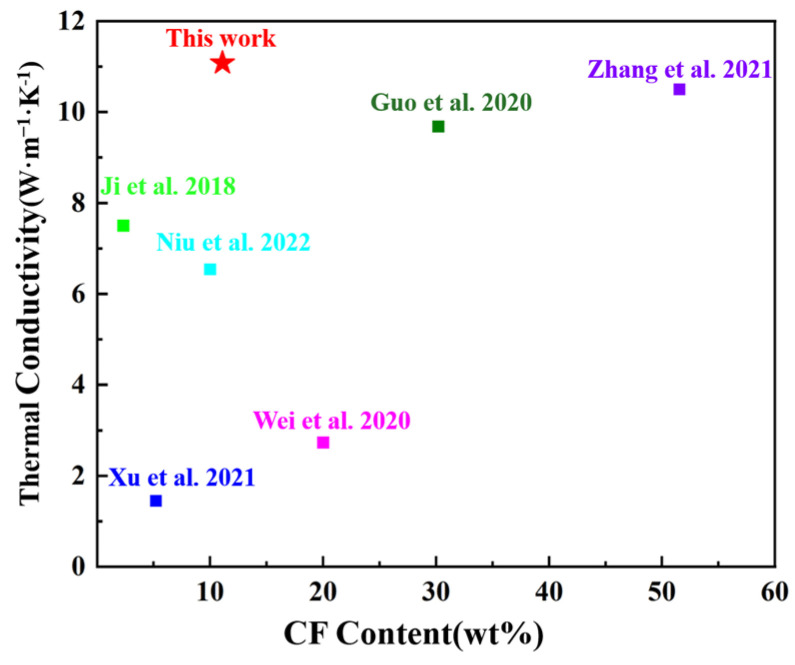
Comparison of thermal conductivities of composites using CF as a thermal conductive filler [18,25,26,27,28,29].

The comparison of the thermal conductivities and CF filling amounts of the composites prepared with CF as a filler is shown in Figure 7. The influence of the matrix due to the low weight ratio is very limited. The filling amount and the filling state of CF are the key factors affecting the thermal conductivity. In this paper, BN was used to provide support for CF and increase thermal conductivity nodes so that the composite material had a high thermal conductivity under the condition of a low CF filling amount. This provides a feasible method for the preparation of high-performance and low-cost TIMs [18,25,26,27,28,29].

## 4. Conclusions

Drawing inspiration from natural leaf and branch structures, this study developed a high-thermal conductivity composite with an oriented structure, which was achieved by minimizing gaps in extrusion molding. CF, exhibiting ultra-high thermal conductivity, serves as the branches, providing efficient thermal conduction channels. Simultaneously, BN platelets, notable for their substantial in-plane thermal conductivity, act as the leaves, offering crucial support to the branches and functioning as nodes within the thermal conduction network. Remarkably, a thermal conductivity of 11.08 W/(m·K) was attained with a CF content of 11.1 wt% and a 1.86 g/cm^3^ density. This research demonstrates the effective combination of 1D and 2D materials to fabricate TIMs with superior thermal conductivity. The simplicity and accessibility of the preparation method underscore its potential for broad application in enhancing the anisotropic properties of TIM materials.

## Figures and Tables

**Table 1:** Composition table for different groups.

Experimental Group	Mass of Component (g)				Content of CF
	SIR	BN	CF	Alumina	
Matrix	12	/	/	/	0
Random-BN	12	12	/	/	0
Oriented-BN	12	12	/	/	0
Random-BN/CF-1	12	12	1	/	4
Oriented-BN/CF-1	12	12	1	/	4
Random-BN/CF-2	12	12	2	/	7.8
Oriented-BN/CF-2	12	12	2	/	7.8
Random-BN/CF-3	12	12	3	/	11.1
Oriented-BN/CF-3	12	12	3	/	11.1
Oriented-CF-1	12	/	1	/	7.7
Oriented-CF-2	12	/	2	/	14.3
Oriented-CF-3	12	/	3	/	20.0
Binder	12	/	/	80	0

## Data Availability

Data are contained within the article.
